# Psychedelic Therapies at the Crossroads of Trauma and Substance Use: Historical Perspectives and Future Directions, Taking a Lead From New Mexico

**DOI:** 10.3389/fphar.2022.905753

**Published:** 2022-06-27

**Authors:** Snehal R. Bhatt, Maya Armstrong, Tassy Parker, Marcello Maviglia, Rebecca Kass, Lawrence Leeman, Paul Romo, Douglas Ziedonis

**Affiliations:** ^1^ Department of Psychiatry and Behavioral Sciences, University of New Mexico, Albuquerque, NM, United States; ^2^ Department of Family and Community Medicine, University of New Mexico, Albuquerque, NM, United States; ^3^ College of Nursing, University of New Mexico, Albuquerque, NM, United States; ^4^ College of Population Health, University of New Mexico, Albuquerque, NM, United States; ^5^ Center for Native American Health, University of New Mexico, Albuquerque, NM, United States; ^6^ American Indian Health Research and Education, University of New Mexico, Albuquerque, NM, United States; ^7^ Executive Vice President, University of New Mexico Health Sciences Center, Albuquerque, NM, United States

**Keywords:** PTSD, psychedelics, adverse childhood experiences, social determinants of health, AI/AN health, substance use

## Abstract

Post-traumatic stress disorder (PTSD), a common condition with potentially devastating individual, family, and societal consequences, is highly associated with substance use disorders (SUDs). The association between PTSD and SUD is complex and may involve adverse childhood experiences (ACEs), historical and multi-generational traumas, and social determinants of health as well as cultural and spiritual contexts. Current psychosocial and pharmacological treatments for PTSD are only modestly effective, and there is a need for more research on therapeutic interventions for co-occurring PTSD and SUD, including whether to provide integrated or sequential treatments. There is a current resurgence of interest in psychedelics as potential treatment augmentation for PTSD and SUDs with an appreciation of the risks in this target population. This paper reviews the historical perspective of psychedelic research and practices, as well as the intersection of historical trauma, ACEs, PTSD, and SUDs through the lens of New Mexico. New Mexico is a state with high populations of Indigenous and Hispanic peoples as well as high rates of trauma, PTSD, and SUDs. Researchers in New Mexico have been leaders in psychedelic research. Future directions for psychedelic researchers to consider are discussed, including the importance of community-based participatory approaches that are more inclusive and respectful of Indigenous and other minority communities.

## Introduction

New Mexico is one of the most ethnically diverse states in the continental United States, with 49.3% of the population identifying as Hispanic, 11% identifying as American Indian/Alaska Native [AI/AN], and 36.8% as non-Hispanic white (per the U.S. Census Bureau report in 2020–2021 estimates). According to the census bureau, New Mexico is the 11^th^ most diverse state in the nation, with a diversity index of 63.0%. New Mexico’s cultural diversity includes 23 tribal nations (19 Pueblos, three Apache tribes, and the Navajo Nation) and many subcultures within the Hispanic population (e.g., country of origin, region within the state), each with its own rich history, cultural traditions, and linguistic and dialectal diversity. New Mexico is also one of the most rural states in the US (5th largest state by geographic size and 36th by population—only 2.08 million). New Mexico has pockets of wealth and many points of pride; however, overall it has a challenged economy with high unemployment, poverty, and illiteracy rates.

New Mexican communities have experienced severe consequences of substance abuse, which is one of the state’s leading causes of death (National Institute on Drug Abuse, 2020). The negative consequences of excessive substance use and other social determinants of health (SDOH) in New Mexico are not limited to death but also include adverse childhood events (ACEs), domestic violence, crime, poverty, and unemployment as well as morbidity related to liver and heart disease, injuries, and a variety of other medical problems (New Mexico Department of Health, 2021). Furthermore, over the period of 1981 through 2019, New Mexico’s suicide rate was consistently among the highest in the nation, at 1.5 to 1.9 times the national rate (New Mexico Department of Health, 2021).

Post-traumatic stress disorder (PTSD) is commonly associated with substance use disorders (SUDs) and worsens the course and outcomes of SUDs for the individual, family, and communities. The complex association between PTSD and SUD is important to understand in relation to the role of ACEs, historical and multi-generational traumas, and SDOH as well as cultural and spiritual contexts.

A closer look at the New Mexico state-specific data reveals some alarming trends. For example, AI/AN and Hispanic populations have the highest rates of alcohol-related death and chronic disease. AI/AN populations have the highest rates of suicide as well as alcohol-related injury and traffic fatalities, while Hispanic populations have the highest rates of fatal drug overdose (New Mexico Legislative Finance Committee, 2019; New Mexico Department of Health, 2021), all of which may be categorized under the term, “diseases of despair.” One of the northern New Mexico counties with a minority-majority population has received national attention due to having one of the highest rates of overdose mortalities in the nation. Between 2015 and 2019, this county reported alcohol-related death rates greater than four times the national average and fatal drug overdose occurring at close to four times the national rate (New Mexico Workforce Connection, 2019).

Finally, it should be mentioned that veterans account for a higher proportion (8.4%) of New Mexico’s population compared with the national average of 6.9%. Nationally, rates of PTSD among veterans are higher than the general population, with up to 17% of veterans from the Iraq/Afghanistan conflicts reporting current PTSD [4, 5].

One purpose of this paper is to highlight the role of New Mexico in the history of psychedelic research and to propose guidelines for future psychedelic research related to PTSD and SUD that are more inclusive and respectful of Indigenous and other minority communities. We begin by highlighting the impact of ACEs and multigenerational and historical trauma on the development of PTSD and SUDs (focusing on the experience in New Mexico) and by emphasizing the urgent need for new treatments for these highly morbid conditions. Next, we review relevant research on psychedelics and related compounds for treatment of various conditions, including PTSD and SUDs and highlight the role played by New Mexico in this research. Finally, we suggest an approach for further research guided by our experiences in New Mexico and based on a community-based participatory research (CBPR) approach in which Indigenous and other minority communities are involved as equal partners. CBPR is intended to accommodate and, indeed, privilege the multiple worldviews, beliefs, spiritual preferences, and traditional practices of our community research partners in the development or advancement of science that is of direct benefit to those communities. With authentic engagement communities and individuals can become receptive to healing from a history of trauma (Munro et al., 2017).

### The Role of ACEs

SUDs and substance misuse are driven by complex underlying issues, such as genetics, SDOH, ACEs, and trauma, which often cross generations. Indeed, per the most recent census report, 18.2% of New Mexicans live below the poverty level, but this may be significantly underestimated. According to data from the Children, Youth, and Families Department (CYFD), between 2014 and 2018, substance use was a factor in 64% of substantiated cases; parental substance use was associated with a 2-fold likelihood of children being removed from their homes. On a related note, the 2017–2018 National Survey of Children’s Health demonstrated that 27% of New Mexican children have experienced two or more ACEs, compared to 19% of children nationwide. An estimated 67.6% of NM adults reports at least one ACE, while nearly one in four report four or more ACESs (New Mexico Dept. of Health, 2021). Among incarcerated youth, a staggering 86% had experienced four or more ACEs and 96% struggled with SUDs (New Mexico Legislative Finance Committee, 2019).

There exists a strong dose-dependent link between ACEs and multiple physical and mental health co-morbidities, including depression, anxiety, and severity of PTSD (Felitti et al., 1998). Having four or more ACEs has been shown to significantly impact multiple health domains, including physical inactivity, obesity, diabetes, smoking, heavy alcohol use, poor self-rated health, cancer, heart disease, respiratory disease, mental illness, and problematic drug use, along with interpersonal and self-directed violence (Hughes et al., 2017). The relationships among ACEs, mental health conditions, and unhealthy substance use are well-established but complex and incompletely understood. In addition to cumulative effects of multiple categories of ACEs, age at exposure and duration of exposure appear to influence outcomes (Campbell et al., 2016; Shin et al., 2018; Rhee et al., 2019). Furthermore, the clustering of ACEs (exposure to one category of ACE increases risk for exposure to other categories), the development of risky behaviors including substance use, and the increased risk for homelessness (Ararso et al., 2021) seem to ensure the perpetuation and escalation of adverse effects.

### Impact of Multigenerational and Historical Trauma on AI/AN and Hispanic Communities in New Mexico

Historical trauma is defined as “cumulative emotional and psychological wounding across generations, including the lifespan, which emanates from massive group trauma” (Brave Heart, 2003). Importantly, and perhaps unsurprisingly, historical trauma is strongly related to the experience of lifetime traumatic events as well as to the development of conditions such as unresolved grief, complicated/prolonged grief, PTSD, depression, and substance abuse (Heart et al., 2011).

As mentioned, New Mexico is home to 23 Tribes, Pueblos, and Nations. And while all tribes have unique histories and unique cultural and spiritual traditions, they are united in their shared experiences of colonization, forced removal from traditional lands, suppression of traditional spiritual and cultural practices, and forced assimilation through boarding school placements and forced relocations. Literature has indeed focused on the etiologic role of historical trauma as a factor in the high rates of SUDs in New Mexico. It has been proposed that a lack of validation of the grief associated with this collective generational trauma, combined with ongoing discriminatory policies and modern traumatic losses, contribute to historical trauma responses, which can include depression, PTSD symptoms, anger, and self-destructive behaviors including substance use and suicide (Goodkind et al., 2012).

While this concept has been extensively studied within AI/AN populations, it likely applies to other communities of color that historically have experienced race-related physical and emotional violence over multiple generations. Hispanic communities of New Mexico, especially in northern New Mexico, are unique in that they predate the colonization of the region by American settlers. It has been proposed that these communities were, in fact, also “colonized,” with resultant loss of traditional ways of life, including forceful annexation of communal land grants, which were central to the social and economic organization of these communities. It has been proposed that these losses have resulted in “cultural PTSD,” which is superimposed with more classically-defined physical and emotional traumas, and may be an important factor leading to the high rates of SUDs in these communities (Baez, 2012).

It must also be pointed out that Indigenous and other minority communities continue to demonstrate tremendous resilience in the face of historical and multigenerational trauma. Many indigenous communities have developed their own traditional therapeutic interventions, which have shown to be very effective in addressing issues such as substance misuse and trauma. However, these interventions are usually focused not on DSM diagnoses but on a holistic concept of well-being and health, emphasizing a strong connection to ancestral lands and revival of cultural and spiritual traditions, and potentially involving an entire community as the agent of healing (Heart et al., 2011; Jacob, 2013).

### Current Treatments for Post-Traumatic Stress Disorder and Substance Use Disorders

It is essential to consider the roles of SDOH, ACEs, and historical trauma, along with culture and traditional healing practices in assessing and treating PTSD in the context of SUDs and vice versa. Therefore, prevention, as well as psychological, pharmacological, and community-based interventions, must be viewed as part of a contextual approach.

Sertraline and paroxetine, both selective serotonin reuptake inhibitors (SSRIs), are approved by the FDA for treatment of PTSD. However, they have modest effects on symptom reduction, with an estimated 40–60% of patients not responding to these treatments (Stein et al., 2006). Perhaps more important are the non-pharmacologic therapies that form the basis of trauma-informed care, which is a model of care delivery that involves understanding, recognizing, and responding to the effects of all types of traumas. This approach emphasizes physical, psychological, and emotional safety while helping survivors rebuild a sense of control and empowerment. It is recommended that trauma-informed care form the backbone of treatment of individuals with histories of trauma and/or PTSD.

Evidence-based manualized trauma-focused psychotherapies, including prolonged exposure, eye movement desensitization and reprocessing (EMDR), cognitive processing therapy, brief eclectic psychotherapy, narrative exposure therapy, and written narrative exposure, are considered the first line of PTSD treatment (Author anonymous, 2017). However, even with these approaches, dropout rates are high, and many patients continue to be symptomatic (Bisson et al., 2013; Watkins et al., 2018).

Even without considering concurrent mental health diagnoses, the treatment of unhealthy substance use remains an enormous challenge with staggering public health implications. Delayed diagnosis, inadequate treatment options, and poor access to evidence-based treatments contribute to low rates of sustained abstinence and high rates of morbidity and mortality (Willenbring, 2014; Blevins et al., 2018; Harris et al., 2020; Olfson et al., 2021).

With respect to co-occurring PTSD and SUDs, there is consensus in the literature that must be treated in an integrated rather than sequential manner, while also addressing the underlying contributing factors, such as ACEs or SDOH. Literature also supports that treating co-occurring PTSD and SUD using exposure-based interventions is safe, acceptable, and effective, though more studies are needed. An example of a specific integrated approach for co-occurring PTSD and SUD is *Seeking Safety*, a 24-session therapeutic intervention that includes topics such as help-seeking, community engagement and self-soothing. However, use of the *Seeking Safety* curriculum has shown only moderate benefit on PTSD symptoms and has not been shown to improve rates of abstinence (Flanagan et al., 2016; Charney et al., 2018). A Cochrane report of 14 randomized clinical trials (1506 participants) found little evidence to support the use of *non*-exposure-based interventions among patients with co-occurring SUD and PTSD. The authors noted that the review is limited by low quality studies and high attrition rates, and more rigorously designed trials are clearly needed (Bisson et al., 2015).

As the above review demonstrates, currently available psychosocial and pharmacological treatments for PTSD are only modestly effective, with even fewer options for co-occurring PTSD and SUD. Additionally, it is pertinent to note that, due to low representation in most clinical trials, there is no clear evidence of efficacy for any of these western-based treatments in indigenous populations (Burlew et al., 2011; Greenfield and Venner, 2012; Dickerson et al., 2014). This highlights the need for investigation of new treatment approaches. Recently, attention has turned towards the use of psychedelic substances as a potential treatment for individuals with PTSD and SUDs.

### Recent Trials of Psychedelics for Post-Traumatic Stress Disorder and Substance Use Disorders

In light of the stark treatment gaps and limited treatment options highlighted above, the recent resurgence of interest in psychedelics research has led to a growing interest in the potential applications of psychedelic therapies for treating PTSD and SUDs. Classic psychedelics are a group of compounds, including lysergic acid diethylamide (LSD), mescaline, psilocybin, and N,N-dimethyltryptamine (DMT), which share a common mechanism of action, mainly through agonism at the serotonin 5-HT2A receptors.

Despite the existence of FDA approved treatments for tobacco use disorders (five types of nicotine replacement therapies, plus varenicline, bupropion, and transcranial magnetic stimulation), alcohol use disorders (naltrexone, acamprosate, and disulfiram) and opioid use disorders (methadone, buprenorphine, naltrexone), use of these substances continues to impose incalculable burden on global health. Psilocybin, administered within a psychotherapeutic framework, has shown promise in the treatment of a variety of substance use disorders. An investigation combining psilocybin with a structured smoking cessation protocol demonstrated abstinence at 6-month follow-up in 80% of participants (Johnson et al., 2014), with 60% of these participants remaining abstinent at long-term follow up of >16 months (Johnson et al., 2017). Indeed, these numbers are considerably higher than those typically achieved with more traditional approaches. Emerging data for psilocybin therapy also suggest a therapeutic role for individuals with alcohol use disorder. In a proof of concept trial of psilocybin assisted therapy for alcohol use disorder, Bogenschutz et al. (2015) reported that psilocybin administration sessions within a context of psychotherapy combining Motivational Enhancement Therapy and therapy designed to prepare for, and debrief from, the psilocybin sessions, led to significant increases in abstinence following psilocybin administration; encouragingly, these gains were maintained at 36 week follow-up. In naturalistic studies, ibogaine has been reported as a specific treatment for opioid use disorder (Brown et al., 2019), but as of this review, this had not been tested in a clinical trial setting. In the 1960s and 1970s, LSD was extensively studied as a treatment for alcohol use disorder, and a meta-analysis of six randomized controlled trials including 536 subjects demonstrated a beneficial effect (Krebs and Johansen, 2012).

While there is limited research into the effects of classic serotonergic psychedelics on PTSD, another compound, 3,4-methylnedioxymethamphetamine [MDMA], which shows some overlap of neurobiological and clinical effects with those of the classic psychedelics, has received breakthrough drug status from the FDA for the treatment of PTSD based upon a series of phase 2 and one phase 3 clinical trial (Feduccia et al., 2019; Mithoefer et al., 2019). Most recently, results from a phase 3 trial funded by the Multidisciplinary Association for Psychedelic Study (MAPS) show robust reduction in PTSD symptoms, even among participants with features normally associated with treatment resistance (Mitchell et al., 2021). In this study, MDMA was found to induce significant and robust attenuation in CAPS-5 scores compared with placebo and to significantly decrease the total score on the Sheehan Disability Scale. Importantly, 67% in the MDMA group no longer met the criteria for PTSD at the 18 week primary study endpoint, compared to 32% in the placebo group. MDMA did not induce adverse events of abuse potential, suicidality or QT prolongation. Such promising findings have led many combat veterans to advocate for rescheduling of MDMA to improve access to treatment. The compound also is being studied in the setting of SUDs, with a recent proof-of-concept study showing reduced alcohol consumption and improved psychosocial functioning in individuals with alcohol use disorder who received an 8 week course of recovery oriented therapy combined with two MDMA sessions following alcohol detoxification (Sessa et al., 2021).

Another agent that, although not classified as a “classic psychedelic” but still with some overlap of clinical effects with those of psychedelics, is ketamine. Studies have demonstrated significant and rapid reduction of PTSD symptom severity following IV infusion of ketamine persisting for up to 7 days after a single infusion (Feder et al., 2014; Pradhan et al., 2017). Ketamine has been investigated as a potential treatment for various substance use disorders. Krupitsky et al. (2007) showed that ketamine assisted psychotherapy is more effective than placebo at promoting abstinence in individuals with heroin addiction. A study by Dakwar et al. (2019) combining a single infusion of ketamine with mindfulness-based therapy showed improved abstinence among individuals with cocaine dependence. Yet another study by Dakwar et al. (2020) showed that a single ketamine infusion combined with a five-week course of Motivational Enhancement Therapy for alcohol use disorder significantly increased the likelihood of abstinence, reduced the likelihood of heavy drinking days, and delayed the time to relapse compared to an infusion of midazolam. Even more recently, Azhari et al. (2021) carried out a proof of concept study showing that ketamine infusions combined with Motivational Enhancement Therapy and Mindfulness Based Relapse Prevention led to reduced cannabis use and increased confidence in the ability to abstain from cannabis in triggering situations. A 2018 review article (Ivan Ezquerra-Romano et al., 2018) focusing on the role of ketamine in the management of a variety of SUDs summarizes clinical data with large effect sizes and presents possible mechanisms of action, including disruption of functional networks, reconsolidation of memories, and rapid antidepressant effects.

### Psychedelics in New Mexico: Historical and Sacramental Use

Classic psychedelics likely have been used by humans from multiple cultures since pre-historic times. The state of New Mexico lies in a region that has housed cultures with long histories of sacramental use of psychedelic compounds. For example, peyote bulbs stored in the southwestern United States have been radiocarbon dated to 3780–3660 BCE (El-Seedi et al., 2005). Indeed, New Mexico is within the natural geographic range of peyote and San Pedro cacti, both containing the alkaloid mescaline. Sacramental use of peyote, protected by the American Indian Religious Freedom Act of 1994, continues to be prevalent among members of the Native American Church (NAC) with an estimated membership of 600,000 individuals (Prue, 2014). Additionally, New Mexico is home to the United States headquarters of Uniao do Vegetal [UDV], whose members use ayahuasca, a psychedelic drink combining N,N-dimethyltryptamine (DMT) and MAO inhibiting harmala alkaloids, as a sacrament. Western scientists have examined the effects of naturalistic use of these sacraments. Peyote has long been examined as a potential treatment of alcoholism among AI/AN populations (Blum et al., 1977; Halpern et al., 2005). A recent online questionnaire (Agin-Liebes et al., 2021) demonstrated that naturalistic use of mescaline was associated with self-reported improvements in psychiatric symptoms and positive life changes. Additionally, multiple studies have associated ritualistic use of ayahuasca with positive effects on substance-related problems, as well as on psychological, spiritual, and physical well-being (Fábregas et al., 2010; Thomas et al., 2013; Barbosa et al., 2018; Malcolm and Lee, 2017; O'Shaughnessy et al., 2021; Argento et al., 2019).

It is important to note that there are differences between the use of psychedelics in a culturally sanctioned sacramental manner, and their use in clinical research settings. Firstly, most clinical trials use isolates of specific active compounds (e.g., psilocybin), whereas traditional healing practices involve whole plant medicines containing a wide variety of chemical compounds, which may interact in various ways (the so-called “entourage effect”). The sacramental use of these plant medicines typically begins well before the administration of the medicine, with ceremonies and prayers associated with harvesting and preparation. Additionally, indigenous systems of healing take a holistic wellness-based approach to the use of these medicines, as opposed to western investigations, which focus on exploring the efficacy of psychedelic compounds for a specific illness or a condition. Moreover, within indigenous contexts, spiritual and mystical experiences along with connectedness to other humans and the environment are considered central to the healing process, and ceremonies often take place in a group setting. This is in sharp contrast to western psychedelic research, which has used an individual therapy model of healing.

Importantly, western science has increasingly begun to explore the role of groups within a psychedelic therapy context. A recently published trial of psilocybin-assisted therapy for the treatment of demoralization in men with AIDS was promising with respect to feasibility and efficacy. In that trial, group therapy sessions preceded and followed individually administered psilocybin sessions. The group format not only decreased the number of therapist-hours (and therefore cost) but also was associated with a good safety profile and strongly positive outcomes. Although this study did not compare group therapy to individual therapy, the authors were optimistic about “group therapy’s unique capacity to address social isolation, shame, and stigma” (Anderson et al., 2020). Oehen and Gasser (Oehen and Gasser, 2022) have recently published on their therapeutic model combining MDMA and LSD with group therapy in treatment of trauma related disorders.

### Psychedelics and New Mexico—Role in Research

Formal western psychedelic research in New Mexico can be traced back to work by The Association for the Responsible Use of Psychedelic Agents (ARUPA). *Arupa* is a Sanskrit word meaning “formless;” indeed, the organization had no formal structure. It organized invitation-only conferences exploring the therapeutic use of psychedelics at the Esalen Institute in Big Sur, California. Among the attendees were Drs. George Greer and Rick Strassman—both of whom would go on to play seminal roles in modern psychedelic research.

Dr. Greer attended one such six-week conference as a medical student, where he was inspired by Dr. Stanislov Grof, the Czech-born psychiatrist who had carried out important work with LSD and developed holotropic breathwork, a system of achieving non-ordinary states of consciousness without the use of any psychedelic compounds. Another colleague of Grof, Joan Halifax, who co-authored “The Human Encounter with Death” with Grof in 1977, went on to found the Upaya Zen Center in Santa Fe, NM, in 1990. The Upaya Zen Center continues to offer workshops on various topics, including compassionate end-of-life care.

Following medical school, with encouragement from Ralph Metzner (famous for his LSD research at Harvard with Timothy Leary and Richard Alpert), Greer began to offer ketamine to his patients in a therapeutic context. Next, after learning from the famed underground MDMA therapist Leo Zeff, Greer began to treat patients with MDMA-assisted psychotherapy using MDMA manufactured by Alexander “Sasha” Shulgin. Indeed, some of the earliest clinical work with MDMA was carried out by Greer and his spouse, Requa Tolbert, a nurse in Santa Fe, NM. Together, they carried out roughly one hundred MDMA therapy sessions. Their focus was not on a specific clinical condition; rather, they worked with the “worried well,” noting improved communication patterns amongst couples following the sessions. This work led to one of the earliest papers describing the therapeutic potential of MDMA (Greer and Tolbert, 1986). Along with Leo Zeff, Ralph Metzner, Stan and Christina Groff, and many others, Greer and Tolbert helped lay the foundation for the MDMA-assisted psychotherapy model that now is being pursued in ongoing clinical trials.

Some essential elements that Greer helped pioneer include a focus on physical and psychological safety within a framework of psychedelic psychotherapy. This includes a very careful informed consent and peer review process, an inner-directed approach to psychotherapy, and the importance of integration in the healing process (Passie, 2018; Greer, 2020). Additionally, he laid out a rationale for why MDMA-assisted psychotherapy may be beneficial in PTSD, suggesting that the medication allows patients to tolerate fear and anxiety, and to effectively communicate “normally repressed ideas, memories, beliefs, opinions, and attitudes about themselves and others” (Testimony of George Greer, 1984).

Greer and colleagues later went on to found The Heffter Research Institute in 1993, and he continued to serve as the medical director and president until recently. The Institute was founded with a goal of rigorously evaluating scientific projects and securing funding for future psychedelic research. To date, Heffter has provided funding for numerous investigations involving psychedelics, including psilocybin for treatment of cancer-related emotional distress, depression, OCD, and SUDs as well as the relationship between psychedelic use and spirituality and basic science research into brain activity, cognition and behavior.

Dr. Rick Strassman, who also attended an ARUPA conference, is another prominent New Mexican figure in the resurgence of psychedelic research. As a faculty member of the Department of Psychiatry at the University of New Mexico, Strassman was reportedly inspired to study non-ordinary states of consciousness by his experiences with meditation. His early research with psychedelics focused on dose-response and toxicology studies of DMT. In one randomized, double-blind, placebo-controlled study, he administered DMT intravenously to eleven experienced psychedelic users and tracked peak blood concentrations, subjective effects, physiological parameters, and hormone levels (Strassman et al., 1994; Strassman and Qualls, 1994; Strassman, 1995; Strassman et al., 1996). While his early studies of DMT did not explore its use in the treatment of psychiatric conditions, they helped form a framework that contributed greatly to the resurgence of psychedelic research. Importantly, Strassman developed and validated the Hallucinogen Rating Scale (HRS) that continues to be used in clinical psychedelic research (Strassman et al., 1994). He also wrote a best-selling book exploring the effects of DMT (Strassman, 2000). It should be noted that Dr. Strassman has continued to play an important role as a mentor to psychedelic investigators around the United States and internationally to present day, and notably has been a co-author on studies evaluating ketamine-assisted psychotherapy for heroin addiction and psilocybin-assisted psychotherapy for alcohol use disorder (Bogenschutz et al., 2015).

The aforementioned pilot study evaluating the safety and feasibility of psilocybin-assisted treatment for alcohol addiction was carried out at the University of New Mexico by Dr. Michael Bogenschutz (Bogenschutz et al., 2015). In this study, 10 participants with alcohol dependence were treated with psilocybin in addition to 12 weekly psychosocial treatment sessions. Psilocybin was administered at weeks 4 and 8. There were no significant treatment-related adverse events. Abstinence rates, stable during lead-up to the psilocybin sessions, did not increase significantly in the first month of treatment (when participants had not yet received psilocybin), but increased significantly following the initiation of psilocybin treatment. There were strong correlations (r > 0.8) between the intensity of the experience in the first psilocybin session (at 4 weeks)- as measured by the Mystical Experiences Questionnaire [MEQ], the Hallucinogen Rating Scale [HRS] intensity score, and the Altered States of Consciousness summary score and increased abstinence during weeks 5 through 8. The intensity of the week 4 experience also was highly correlated with decreases in craving and increases in abstinence self-efficacy during week 5, suggesting possible mechanisms of action. Currently, a phase 2 multi-site trial powered to evaluate the efficacy of psilocybin for alcohol use disorder is well underway, with the University of New Mexico serving as one of the sites. Lastly, Bogenschutz and Strassman have both engaged in research evaluating the safety of long-term sacramental use of ayahuasca within members of UDV in New Mexico (Barbosa et al., 2009; Barbosa et al., 2012; Barbosa et al., 2016).

## Discussion

### A Potential Paradigm Shift

While much remains to be learned about how these psychedelics affect the brain and how these effects may be harnessed effectively to treat individuals with a variety of distressing psychologic conditions, including PTSD, research is pointing towards a variety of plausible mechanisms. It has been shown that psychedelics can induce neuronal plasticity (Ly et al., 2018), enhancing the brain’s ability to adapt, change, and learn. Psychedelics also disrupt the connectivity and activity within the default mode network [DMN], a brain region normally activated when individuals are focused on their internal mental-state processes, such as self-referential processing, introspection, autobiographical memory, or imagining the future. Importantly, aberrant activity and functional connectivity of the DMN has been implicated in a variety of mental health disorders, from depression to post-traumatic stress disorder (PTSD) to alcoholism and other substance use disorders (SUDs). It has been shown that a decrease in resting state functional connectivity within the DMN brought about by LSD, a classic psychedelic, led to a less ruminative and more present-focused and mindful mental state (Speth et al., 2016). This disruption of DMN, combined with insight oriented and emotional breakthrough experiences facilitated by psychedelics, may lead to psychological flexibility, greater openness, a sense of well-being, and enhanced life satisfaction (Griffiths et al., 2008; Roseman et al., 2019; Davis et al., 2021), which may be central to the possible role of classic psychedelics in treatment of PTSD. Psychedelics have also been shown to increase positive affect (Kraehenmann et al., 2015), to increase emotional empathy (Pokorny et al., 2017), and to promote mystical experiences, leading to a greater sense of “oneness” and connectedness, which may serve to counteract the avoidance, loneliness, and isolation, which are central parts of pathology of not just PTSD, but all diseases of despair. By bringing about a “pivotal mental state” (Brouwer and Carhart-Harris, 2020), a hyper-plastic state which can lead to rapid and deep learning and psychological transformation, psychedelics may promote post-traumatic growth (both neurologic and psychologic). Thus, the emerging understanding of possible mechanisms of psychedelic-facilitated healing raises the very exciting paradigm of being able to treat PTSD and SUDs simultaneously and in a holistic manner, addressing the root causes of these conditions to facilitate meaningful transformation. It is important to consider the possibility that this paradigm of healing may serve as a bridge to traditional and indigenous modalities of healing globally through its emphasis on connectedness, spirituality, a holistic concept of well-being, and inner-directed process of healing (Legha and Novins, 2012; Venner et al., 2018; Skewes et al., 2019).

### Safety Considerations

Classic psychedelics have the lowest physiological toxicities of all well-known “drugs of abuse” (Gable, 2004). A recent population-based study of 135,095 randomly selected US adults, including 19,299 users of psychedelics (Johansen and Krebs, 2015) found no significant associations between lifetime use of classic psychedelics and past-year incidence of serious psychological distress, mental health treatment, depression, anxiety, or suicidal ideation or suicide attempts. Despite their schedule I status, classic psychedelics do not reliably lead to self-administration behavior in laboratory animals. In fact, rhesus monkeys found LSD to be aversive (Hoffmeister, 1975).

Until relatively recently, the prevalence of classic psychedelic use in adolescents and young adults appeared to remain relatively stable over time (Miech et al., 2017). However, recent analyses suggest increases in use of LSD (Yockey et al., 2020) and ketamine (Palamar et al., 2021), likely due in part to increased media coverage about potential benefits. Young adults struggling with mental health concerns may be particularly eager to experiment with psychedelics, perhaps in lieu of more traditional pharmacologic and psychologic counseling (Han et al., 2022). Therefore, despite the generally good safety profiles of these agents, further exploration of potential risks is warranted, especially given that most people currently using psychedelic agents are doing so without the guidance of medical or mental health professionals.

All well-described psychedelic compounds are associated with a number of transient effects—both physiologic (e.g., nausea, headache, increases in blood pressure, heart rate, and body temperature) and psychologic (e.g., depersonalization, which may be therapeutic or may be anxiety-provoking). In a safe and supportive setting and guided by trained professionals “bad trips” may be leveraged toward psychospiritual growth, but a less favorable set and setting may lead to significant distress and potential safety issues. Data from the Compass Pathways psilocybin study, which focused on treatment-resistant depression, provide support for overall safety, but some patients did report new or worsening suicidal ideation after treatment [https://compasspathways.com/wp-content/uploads/2021/11/COMP001_-_topline_data.pdf.]. The psychotomimetic effects of psychedelics are precisely what brought them to the attention of early clinicians and researchers, and concern remains about use by individuals with histories of psychosis, who generally have been excluded from clinical trials (Johnson et al., 2008).

It also should be mentioned that one particular psychedelic agent, ibogaine, has been associated with sudden cardiac death when used in nonmedical settings. Administration of ibogaine may result in with QTc prolongation in as many as 50% of users (Knuijver et al., 2022); prolonged QTc is associated with increased risk for potentially fatal torsades de pointe. Furthermore, data on potential drug-drug interactions (including those with other psychoactive medications) are limited. One concerning observation is the apparent increased risk for seizures with use of LSD or psilocybin by individuals on lithium therapy (Nayak et al., 2021).

Although published data on the sacramental use of psychedelics within indigenous cultures has been limited, Halpern et al. (Halpern et al., 2005) demonstrated that among the Navajo, members of NAC who regularly use peyote had no significant psychological or cognitive deficits compared to their counterparts who did not regularly use peyote. Additionally, lifetime peyote use was not associated with impaired neuropsychological performance. Multiple studies have looked at long-term outcomes in regular users of ayahuasca and have reported no persisting adverse effects on neuropsychological functioning (Bouso et al., 2012; Barbosa et al., 2016). Pertinently, there are no data to suggest that sacramental use of psychedelics within indigenous communities may contribute to higher rates of substance use disorders.

While psychedelics certainly can be misused and may carry risks when used recreationally, extensive clinical research with the classic psychedelics has established their relative safety within research settings when subjects are carefully screened, supervised, and followed. Guidelines have emerged that provide “best practices” for safely conducting research with psychedelics (Johnson et al., 2008), and risks for anxiety and transient psychotic reactions may be mitigated through participant screening, education, close supervision and monitoring by trained staff. It also must be pointed out that when conducting research in partnership with Indigenous individuals and communities, the importance of contextual factors cannot be neglected, since these appear to be pivotal to positive outcomes.

### Future Directions

The term “psychedelic renaissance” is being used to describe the current resurgence in research focusing on psychedelic compounds and their potential therapeutic applications. It is an apt term not only for its etymologic meaning, “rebirth,” but also in its recapitulation of that transitional historic period between the medieval ages and modern times, when exploration and discovery focused not only on geography but science, art, and literature. Indeed, psychiatry has been in need of such growth and renewal, if not a full-blown paradigm shift, especially with respect to complex and difficult-to-treat conditions that have defied simple mechanistic explanations and therapeutic approaches. The spark of psychedelic therapies has ignited curiosity not only in the neurobiology of conditions such as depression and PTSD but also in the mechanisms underlying the therapeutic effects of these “mind-manifesting” compounds and ultimately in larger questions about consciousness, traditionally explored more by philosophers than scientists.

The scientific understanding of the mechanisms through which psychedelic compounds act has moved from superficial relationships between receptors and neurotransmitters to grappling with the far more complex concept of neuroplasticity. Happily, our current and rapidly evolving technologies for functional imaging and single cell RNA sequencing are allowing much more detailed exploration of the pathologic and therapeutic processes at play. How are these mechanisms and processes different with psilocybin vs*.* MDMA vs*.* ketamine? Which manifestations of trauma or which subtypes of depression are best suited to treatment with a specific type of compound supported by a particular therapeutic context? Much remains to be explored in this psychedelic renaissance. As we move forward as a scientific community, we must not only remain vigilant so as not to allow our enthusiasm to outpace our knowledge, but we must also continue to reflect on the traditional contextualized use of psychedelic compounds for growth and healing and to provide respectful and adequate tribute (in philosophic, intellectual, and financial terms) to those cultures who acted as their custodians for eons.

There is indeed a need to engage indigenous and other minority individuals, communities, and scholars in working together on trying to develop and evaluate innovative treatments for PTSD and SUD, including psychedelic therapies and research. A recent study (Michaels et al., 2018) evaluating 17 psychedelic studies published between 2000 and 2017 reported that 82.5% of participants in these studies were non-Hispanic white; AI/AN populations accounted for only 4.7% of participants. Not only does this sampling bias limit the generalizability of the results, but it also precludes opportunities for understanding the interplay of factors such as cultural and historic traumas, microaggressions, and discrimination on the development of PTSD and the response to psychedelic treatments.

These low rates of participation by individuals from multiple racial and ethnic minority groups have also been shown in non-psychedelic research. A recent paper (Vigil et al., 2021) showed that AI/AN individuals accounted for only 1% of all participants in NIH- Intramural Research Program clinical studies. This study identified that only 21% of studies included at least one AI/AN participant, and 41% of these studies included only one AI/AN participant. In order to increase AI/AN research participation, there must be authentic engagement with the community, with opportunities for bi-directional learning and acknowledgment of community wisdom and knowledge. Practical issues such as realistic incentives (especially if participants have to travel long distances and in inclement weather), consideration for childcare or providing childcare, informed consents that are written in plain language, providing information about research results to every extent possible, using clinical trial approaches that don’t violate cultural values (such as ensuring that participants in all study conditions receive potentially beneficial interventions) must be considered. Peteriet et al. (Petereit and Burhansstipanov, 2008) provide a pragmatic set of approaches for securing and maintaining trust with AI/AN communities during clinical trials, and report on their experience in getting permission to conduct a genetic study.

Efforts to increase AI/AN participation in psychedelic research specifically must involve a careful, clear articulation of how potential benefits of the research outweigh the potential risks. For someone who has used a particular substance in traditional spiritual manner, the approach of Western science to confirm its efficacy whereby the substance is measured and manipulated in other ways is a foreign and troubling concept because it has the potential for diminishing the traditional spiritual and healing properties. Therefore, meaningful dialogue should proceed the study design. Efforts must be made for our protocols to include language that calls for a focus on community-defined research benefits, diversity, authentic engagement of communities of color, including Indigenous and other minority communities, ensuring diversity within the research team and study therapists. People working in the environments in which therapy takes place need to be welcoming, knowledgeable and respectful of the cultures, and not make assumptions about others’ needs. Additionally, focus must be maintained not just on the individual, but on the community. Finally, the concept of the sacred must be preserved.

The authors of this manuscript advocate for research approaches such as community-based participatory research (CBPR), that depend on community advisory structures and that include service-users and their families in all stages of design and implementation (Smikowski et al., 2009). We strongly believe that the CBPR framework has the potential for encouraging indigenous and other minority groups’ participation in all the salient aspects of psychedelic research.

Federal agencies such as NIH can help improve AI/AN participation in psychedelic research. NIH and other federal agencies have the very important role of preventing stigmatization of Indigenous populations as a consequence of research. In addition, grant development must include a built in period of time for relationship building activities that is realistic. An important long-term priority must be to train more Indigenous scientists and to recruit and retain them in academic institutions. AI/AN psychedelic researchers must be protected in their career development and be given recognition in the tenure process for community-based research development and there should be incentives for this particular type of work such as NIH payback of student loans. Like the communities that AI/AN researchers come from, they need to be reassured of the benefits of the research, because failure or injury in the process of the psychedelic research may cause estrangement of the AI/AN researcher from their tribal community.

Beyond ensuring diversity of research participants, efforts must be made to respect the indigenous communities and cultures that have long understood the healing potential of these natural compounds and acted as the custodians of the plants, animals, and fungi that produce them. These communities continue to be disproportionately affected by emotional suffering and distress. Through meaningful discussions with our AI/AN leaders and scholars, we have come to believe that scientists must honor the fact that the current surge in psychedelic research would not be possible without traditional knowledge, which historically has been extracted without consent and without consideration of benefit to these same cultures. One recent example is the inclusion of peyote in psychedelic decriminalization efforts in California, despite opposition from indigenous groups who use this critically endangered species in their spiritual practices and are rightfully concerned about its exploitation [https://chacruna.net/native-american-statement-regarding-decriminalization-of-peyote/]. Another recent example surrounds the future of psilocybin therapeutics. Psilocybin is derived from species of mushrooms that have been used sacramentally by Mazatec people for generations. There currently are at least 24 patents related to psilocybin, yet there are no recorded plans of consultation, reciprocation or compensation from profits generated by these patents (Gerber et al., 2021). It has been proposed that such a system of knowledge extraction has ironically led to further trauma in these communities, even as these compounds are explored for treatment of PTSD. Importantly, these actions may in fact violate the United Nations Convention for Biological Diversity as well as the United Nations Declaration on the Rights of Indigenous Peoples, which provide indigenous peoples the right to protect, preserve, and develop traditional knowledge, and to benefit from their development (Fotiou, 2020; George et al., 2020; Gerber et al., 2021).

There is a need to better understand how to link public health, clinical services, and communities in addressing SDOH, ACEs, and historical trauma in improving the health of individuals, families, and communities, in addition to new approaches to the treatment of PTSD and SUD. This work will help us better understand community needs and their ideas for solutions related to these health matters, and their views, concerns, and hopes for using- or not using-psychedelics in treating PTSD and SUD, and with what psychosocial treatments, including twelve-step programs, other mutual support programs, family and network-based interventions, and culturally based interventions.

Within New Mexico, as we work to advance treatment for PTSD and SUD, we hope to call for an approach to research that begins with self-awareness and cultural humility. At the University of New Mexico, we have a rich history of community based participatory research that has helped increase cultural inclusiveness, but more must be done. The tenets of “Relevance,” “Authentic Participation,” “Trust,” “Cultural Respect,” and the honoring of “Sovereignty (self-governance) and Self-Determination” must be kept sacrosanct in all aspects of research process.
